# Demographic and socioeconomic factors influencing the incidence of ankle fractures, a national population-based survey of 512187 individuals

**DOI:** 10.1038/s41598-018-28722-1

**Published:** 2018-07-11

**Authors:** Song Liu, Yanbin Zhu, Wei Chen, Lin Wang, Xiaolin Zhang, Yingze Zhang

**Affiliations:** 1 Department of Orthopaedic Surgery, the Third Hospital of Hebei Medical University, Shijiazhuang, Hebei 050051 P.R. China; 2 Key laboratory of biomechanics of Hebei Province, Shijiazhuang, Hebei 050051 P.R. China; 3 Department of statistics and epidemiology, Hebei Medical University, Shijiazhuang, Hebei 050000 P.R. China

## Abstract

This study aimed to investigate the population-based incidence rate of ankle fracture and associated risk factors in China. All the data on ankle fractures were available from the China National Fracture Survey (CNFS) conducted between January and May in 2015. All eligible household members were sampled from 8 provinces, 24 urban cities and 24 rural counties in China, using stratified random sampling and the probability proportional to size method. Questionnaires were sent to every participant for data collection and quality control was accomplished by our quality control team members. A total of 512187 valid questionnaires were collected and relevant data were abstracted and analyzed. One hundred and ninety patients sustained 193 ankle fractures in 2014, indicating the incidence rate was 37.1 (95% CI, 31.8–42.4)/100,000 person-year. Alcohol consumption, living alone and average sleep time <7 h/d were identified as independent risk factors for ankle fracture in both males and females. Previous history of fracture was identified as significant risk factor of ankle fracture in females but not in males. Therefore, specific public health policies focusing on decreasing alcohol consumption and encouraging individuals to obtain sufficient sleep should be implemented. Individuals living alone should focus more on healthcare, especially in those with previous fracture.

## Introduction

Ankle fracture is the most common injury in the department of emergency and orthopaedics, accounting for 46.7% of tibial/fibula fractures and 7.6% of all fractures^1^. Currently, a number of studies reported the incidence rate and risk factors associated for ankle fracture, both in specified and non-specified patient groups^2–5^. However, most epidemiologic studies only specified a single hospital, a subgroup of patients like elderly and others, or a certain region, which might be limited by the small size sample^2,4,6,7^. And a major problem was the substantial variations in incidence rates reported in literature. By far, the national epidemiological data on incidence rate and associated risk factors for ankle fracture are scarce.

With a population of over 1.36 billion worldwide, China had a substantial diversity in term of economic development, cultural practices, and lifestyles among different regions and ethnic groups. Therefore, we infer the incidence rate of traumatic fractures will be varied based on different settings. Currently, Chinese National Health Services Survey (CNHSS) is the sole epidemiologic database of national level for collection of data on self-reported fractures at 2 weeks before the surgery. Therefore, some less-severe fractures treated by conservative methods might be missed out. In addition, this national survey system only collected the basic data on fractures (e.g. age, gender and fracture occurrence timing), but without any information on bone site, type of fracture, injury mechanism and related risk factor (geographical location, socioeconomic and lifestyles).

Therefore, we designed and performed the China National Fracture Study (CNFS) in 2015 years to investigate the population-based incidence rates and related socioeconomic and lifestyles for traumatic fracture of all body sites. The related results have been published elsewhere, recently^8^. In the present study, data on ankle fractures were extracted from the CNFS database and we had 2 aims: 1, to report the national population-based incidence of ankle fracture in China and 2, to explore the related risk factors in term of demographics, socioeconomics, and lifestyles.

## Methods

The work has been reported in line with the STROCSS (Strengthening the Reporting of Cohort Studies in Surgery) guidelines.

### Sampling method

The entire sampling process of CNFS was completed with combined use of optimum allocation and random stratified and probability proportionate to size (PPS) sampling method. During the first phase, 8 provinces (municipalities) were selected from 31 provinces (municipalities or autonomous regions) in mainland China based on geographical location, socioeconomic development, climate and population size, using stratified random sampling method. And within each targeted province (municipalities), sampling was done separately in urban and rural areas.

For urban areas, using the optimum allocation and random stratified and probability proportional to size method we selected a certain number of streets ranging from one to six in each sampled city, and a range from one to ten neighborhood communities from each chosen street, based on the geographical location from west to east on the electronic map. The total number of families in each neighborhood community was determined by the average number of household members according to the latest official census data. All members of eligible families to be invited to participate in this study must live in their current residence for at least 6 months.

For rural areas, we sampled 1–5 counties in each selected province and then in each county, 1–8 towns were selected. In each town, 1–14 administrative villages were sampled. The sampling process was completed using the probability proportional to size method. In each village, households were calculated and selected based on probability proportional to size principles. All members of eligible families to be invited to participate in this study must live in their current residence for at least 6 months.

### Participants and survey

In principle, eligible household members must be personally interviewed by trained research team members. However, for preschool and primary school children, their information should be provided by their guardians in order for data accuracy. For participants who remained non-contactable after repeated visits, telephone surveys had to be used. For selected household members who refused to participate, an alternative household was randomly selected from the candidate list.

A standardized questionnaire was administered by trained research team member for data collection. The detailed information included age, sex, Chinese ethnic origins, marital status, residence, occupation, lifestyles (smoking, alcohol drinking, tea, coffee, carbonate beverages and daily consumption of meat, protein product, dairy products) for all participants, and age of menopause and the number of births only for women. Individuals who had ankle fractures between Jan 1 and Dec 31 2014, then must answer a more detailed accessory questionnaire regarding the fracture occurrence date and place, fracture site (uni, bi or malleolar), and injury mechanism. In addition, they were asked to provide medical records of their fractures, including radiographs, diagnostic reports, and medical reports. And if these data were not available, the survey team paid to obtain a new radiograph of their reported injured ankle joint at a local hospital.

Eight quality control teams were established (one for each province) to check for the quality of collected data. The CNFS was approved by the Institutional Review Board of the Third Hospital of Hebei Medical University, and written informed consent was obtained from all participants before data collection.

### Definition of variables of interest

Individual was divided into Han ethnicity and others combined. Body mass index (BMI) was calculated as weight divided by the square of height, and was subgrouped based the reference criteria suited to Chinese people: underweight, <18.5; normal, 18.5–23.9; overweight, 24–27.9; obesity, >=28^8,9^. Daily diet and drinking including meat and products, bean products, milk and dairy products, coffee, tea and carbonate beverages was divided into 5 groups based on frequency of consumption: never, always (at least 1 serving per day), often (1/day–1/week), occasionally (1/week–1/month) and seldom (<1/month). Calcium or Vitamin D supplementation was defined as positive if participants acknowledged they received Calcium or Vitamin D or both at least 1 month before the ankle fracture occurrence or during the through 2014. Urbanization was divided into 2 groups: 1, rural area and 2, urban area.

### Statistical analysis

Incidence rates for ankle fractures were estimated for the overall populations and for subgroups such as age, regions, ethnic origins, occupation, education and *et al*., stratified by gender. For unordered categorical variables such as occupation, regions, and ethnic origins, the Chi-square test was used to test the differences. For ordered categorical variables such as age and education level, we entered the related data as a continuous variable into a univariate logistic regression model to test the trend difference.

Case group were defined as adult patients (>=15 years) with ankle fractures in 2014, and control group was defined as adult individuals without fractures of any site in 2014. Univariate Chi-square test was used to investigate the potential correlation between ankle fracture and various potential risk factors. Finally, 2 separate multiple logistic regression models were conducted to explore the independent risk factors for ankle fractures among male and female adults. *P* < 0.05 was set as the statistical significance level. Odd ratio (OR) values and corresponding 95% confidence interval (95% CI) were used to indicate the correlation magnitude between ankle fracture and risk factors. The Hosmer–Lemeshow test was used to examine goodness-of-fit of the final model and a *p* value > 0.05 indicated an acceptable fitness. SPSS 19.0 was used to perform all the analyses (SPSS Inc.,Chicago, Illinois, USA).

## Results

The CNFS was conducted in January to May in 2015. A total of 512187 valid questionnaires were collected and relevant data were abstracted and analyzed. Through the year of 2014, 1763 patients sustained traumatic fractures (1833 fractures). Of them, there were 190 patients with 193 ankle fractures, indicating that the incidence rate of traumatic ankle fracture in China was 37.1 (95% CI, 31.8–42.4) per 100,000 person-year (Table 1).

**Table 1:** National Incidence of ankle fractures among Chinese population by demographic, socio-economic and geographic factors in 2014.

Items	Sample size	Total		Male		Female	
		Case	Incidence (1/100000)	Case	Incidence (1/100000)	Case	Incidence (1/100000)
Age (years)							
0–14	81166	4	9.0 (1.8–17.9)	1	2.7	5	6.2 (0.8–11.6)
15–44	236206	42	35.5 (24.8–46.3)	31	26.3 (17.0–35.5)	73	30.9 (23.8–38.0)
45–64	138533	39	56.4 (38.7–74.1)	42	60.5 (42.2–78.8)	81	58.5 (45.7–71.2)
65–79	48020	11	45.6 (18.6–72.5)	16	67.0 (34.2–99.8)	27	56.2 (35.0–77.4)
80+	8262	3	75.2 (4.96–146.3)	1	23.4	4	48.4 (9.8–95.8)
P-value for trend test	512187	<0.001		<0.001		<0.001	
Ethnicity							
Han nationality	477,508	91	37.6 (29.9–45.3)	90	38.2 (30.3–46.1)	181	37.9 (32.4–43.4)
Other nationalities	34,679	5	28.4 (3.5–53.3)	4	23.4 (4.7–46.4)	9	26.0 (9.0–42.9)
P-value for difference test	512187	0.539	0.334	0.265			
Region							
East	232,998	46	38.5 (27.4–49.7)	52	45.8 (33.3–58.2)	98	42.1 (33.7–50.4)
Central	99,109	20	40.2 (22.6–57.7)	16	32.5 (16.6–48.4)	36	36.3 (24.5–48.2)
West	180,080	30	33.2 (21.3–45.0)	26	29.0 (17.9–40.2)	56	31.1 (23.0–39.2)
P-value for difference test	512,187	0.753		0.125		0.191	
Urbanization							
Urban area	203,101	37	36.1 (24.5–47.7)	44	43.8 (30.9–56.7)	81	43.3 (34.3–52.4)
Rural area	309,086	59	37.6 (28.0–47.2)	50	32.9 (23.8–42.0)	109	33.0 (26.6–39.4)
P-value for difference test	512,187	0.849		0.164		0.401	
Occupation							
Office worker	61,919	14	42.7 (20.3–65.1)	10	34.3 (13.1–55.6)	24	38.8 (23.3–54.3)
Farmer	106,484	23	47.2 (27.9–66.5)	30	51.9 ()	53	49.8 (36.4–63.2)
Manual worker	148,650	33	39.9 (26.3–53.5)	14	21.2 (10.1–32.4)	47	31.6 (22.6–40.7)
Retired	30,366	6	40.4 (8.1–72.7)	9	58.0 (20.1–95.9)	15	49.4 (24.4–74.4)
Unemployed	32,770	9	21.3 (7.4–35.1)	8	21.0 (6.5–35.5)	17	21.1 (11.1–31.2)
Preschool Children	35,581	1	5.1	1	6.2	2	5.6
Students	80,443	6	62.1 (12.4–111.2)	21	90.9 (52.0–129.8)	27	82.4 (51.3–113.5)
Other	15,974	4	44.1 (8.9–87.4)	1	14.5	5	31.3 (3.9–58.7)
P-value for difference test	512,187	0.157		<0.001		<0.001	
Education (Preschool children and students were excluded)							
Illiterate	74,937	18	52.2 (28.1–76.4)	17	42.0 (22.0–62.0)	35	46.7 (31.2–62.2)
Primary school	158,970	33	41.1 (27.1–55.1)	36	45.7 (30.8–60.7)	69	43.4 (33.2–53.6)
Junior high school	121,415	29	47.2 (30.0–64.3)	27	45.1 (28.1–62.0)	56	46.1 (34.0–58.2)
Senior high school or above	40,841	6	27.8 (5.6–50.0)	5	26.0 (3.2–48.7)	11	26.9 (11.0–42.8)
P-value for trend test	396,163	0.363		0.293		0.560	

There were 94 female and 96 male patients, and their average age was 46.7 years (standard deviation, 17.6; range, 4–87). Slip, trip or fall was the most common cause for ankle factures, and resulted in 76.8% (146/190) of the overall injuries, followed by traffic accidents (22, 11.6%), fall from height (12, 6.3%), crushing injuries (9, 4.7%) and blunt force trauma (1, 0.5%) (Table 2). Home and the road (72.1%, 137/190) were the most common places where ankle fractures occurred (Table 3).

**Table 2:** The causal mechanisms for ankle fractures in China in 2014 (n, %).

Injury Mechanism	Children (0–14 years)	Adult (> = 15 years)		Total
		Male	Female	
Traffic Accident	1 (20.0)	10 (10.5)	11 (12.2)	22 (11.6)
Slip, Trip or Fall	4 (80.0)	71 (74.7)	71 (78.9)	146 (76.8)
Fall from Heights	0	7 (7.4)	5 (5.6)	12 (6.3)
Crushing Injury	0	6 (6.3)	3 (3.3)	9 (4.7)
Blunt Force Trauma	0	1 (1.1)	0	1 (0.5)
Sum	5 (2.6)	95 (50.0)	90 (47.4)	190 (100.0)

**Table 3:** The place of ankle fracture occurrence in 2014 (n, %).

Place of fracture occurrence	Children	Adult (> = 15 year)		Total
		Male	Female	
Home	2 (40.0)	29 (30.5)	36 (40.0)	67 (35.3)
Work unit	0	8 (8.4)	7 (7.8)	15 (7.9)
Building site	0	6 (6.3)	0	6 (3.1)
Road	2 (40.0)	34 (35.8)	34 (37.8)	70 (36.8)
Recreation site	0	4 (4.2)	1 (1.1)	5 (2.6)
Others	1 (20.0)	14 (14.7)	12 (13.3)	27 (14.2)
Sum	5 (2.6)	95 (50.0)	90 (47.4)	190 (100)

Table 1 presented the population-based incidence rates of ankle fractures by individual characteristics and regions, for overall population, males and females. There was no significant difference between those of Han ethnicity and all other ethnicities combined, nor was there any significant difference according to geographical region, urbanization or education, either for overall population or any gender (Table 1). Stratified by occupation, students had the highest incidence rate either in males or females and that was 62.1 and 90.9/100,000 person-year, respectively. The difference of incidence rate in females and overall populations approach to significance (P < 0.001; P < 0.001), but was non-significant in males (P = 0.157). Stratified by age, males of 80+ years and females of 65–79 years had the highest incidence rate of ankle fractures (75.2 and 67.0 per 100,000 person-year), and the difference among respective subgroup was statistically significant (P < 0.001)

Table 4 presented the detailed results of univariate analysis using Chi-square test for adults. For males, Han ethnic origin, cigarette smoking, alcohol consumption, living alone, average sleep time <7 h/d and previous history of fracture were significant risk factors for ankle fractures; and living house facing the sun was identified to be a protective factor. For females, age, Han ethnic origin, occupation, alcohol consumption, living alone, average sleep time <7 h/d, previous history of fracture, earlier age of menopause and more births were identified to have significant effect on the occurrence of ankle fractures.

**Table 4:** Detailed results of univariate analysis for variables of interest.

Variables	Males (n = 214596)		P	Females (n = 214964)		P
	Case (%)	Control (%)		Case (%)	Control (%)	
Urbanization			0.584			0.753
Rural area	65 (68.4)	152229 (71.0)		65 (72.2)	151941 (70.7)	
Urban area	30 (31.6)	62272 (29.0)		25 (27.8)	62933 (29.3)	
Age (year)			0.153			0.001
18–44	42 (44.2)	117763 (54.9)		31 (34.4)	117894 (54.9)	
45–64	39 (41.1)	68753 (32.1)		42 (46.7)	69026 (32.1)	
65–79	11 (11.6)	24017 (11.2)		16 (17.8)	23721 (11.0)	
> = 80	3 (3.2)	3968 (1.8)		1 (1.1)	4233 (2.0)	
Bean product			0.481			0.607
Never	0	1388 (0.6)		1 (1.1)	1256 (0.6)	
Always	13 (13.7)	40130 (18.7)		19 (21.1)	40552 (18.9)	
Often	43 (45.3)	99663 (46.5)		35 (38.9)	100770 (46.9)	
Occasionally	28 (29.5)	50185 (23.4)		25 (27.8)	50150 (23.3)	
Seldom	11 (11.6)	23135 (10.8)		10 (11.1)	22146 (10.3)	
Ethnicity			<0.001			<0.001
Han	90 (94.7)	200253 (93.4)		86 (95.6)	200621 (93.4)	
Other	5 (5.3)	14248 (6.6)		4(4.4)	14253 (6.6)	
BMI			0.481			0.123
18.5–23.9	64 (67.4)	138093 (64.4)		54 (60.0)	144340 (67.2)	
24–27.9	21 (22.1)	58184 (27.1)		26 (28.9)	44780 (20.8)	
>=28	6 (6.3)	8363 (3.9)		6 (6.7)	9367 (4.4)	
<18.5	4 (4.2)	9861 (4.6)		4 (4.4)	16387 (7.6)	
Education			0.985			0.923
Illiterate	18 (18.9)	34381 (16.0)		17 (18.9)	40393 (18.8)	
Primary school	35 (36.8)	82327 (38.4)		37 (41.1)	80597 (37.5)	
Junior high school	30 (31.6)	68337 (31.9)		27 (30.0)	66554 (31.0)	
Senior high school or above	12 (12.7)	29456 (13.7)		9 (10.0)	27330 (12.7)	
Occupation			0.981			<0.001
Unemployed	6 (6.3)	9597 (4.5)		21 (23.3)	22993 (10.7)	
Office worker	1 (1.1)	6276 (2.9)		1 (1.1)	6188 (2.9)	
Manual worker	33 (34.7)	82403 (38.4)		14 (15.6)	65762 (30.6)	
Farmer	23 (24.2)	48460 (22.6)		30 (33.3)	57500 (26.8)	
Retired	6 (6.3)	14777 (6.9)		9 (10.0)	15420 (7.2)	
Students	9 (9.5)	17580 (8.2)		5 (5.6)	17253 (8.0)	
Other	17 (17.9)	35508 (16.5)		10 (11.0)	29758 (13.8)	
Meat and product			0.777			0.267
Never	0	29 (0.001)		0	2523 (1.2)	
Always	44 (46.3)	111523 (52.0)		36 (40.0)	104977 (48.9)	
Often	33 (34.7)	65004 (30.3)		33 (36.7)	65151 (30.3)	
Occasionally	15 (15.8)	29111 (13.6)		14 (15.6)	31609 (14.7)	
Seldom	3 (3.2)	8834 (4.1)		7 (7.8)	10614 (4.9)	
Dairy and product			0.212			0.533
Never	38 (40.0)	92035 (42.9)		30 (33.3)	77457 (36.0)	
Always	22 (23.2)	31533 (14.7)		14 (15.6)	38374 (17.9)	
Often	15 (15.8)	34564 (16.1)		24 (26.7)	41654 (19.4)	
Occasionally	13 (13.7)	35378 (16.5)		15 (16.7)	37593 (17.5)	
Seldom	7 (7.4)	20991 (9.8)		7 (7.8)	19796 (9.2)	
Cigarette smoking			0.027			0.569
No	41(43.2)	116858 (54.5)		2 (2.2)	7080 (3.3)	
Yes	54 (56.8)	97643 (45.5)		88 (97.8)	207794 (96.7)	
Alcohol consumption			0.003			<0.001
No	30 (31.6)	100778 (47.0)		65 (72.2)	188566 (87.8)	
Yes	65(68.4)	113723 (53.0)		25 (27.8)	26308 (12.2)	
Living alone			0.004			0.032
No	2 (2.1)	756 (0.4)		88 (97.8)	214208 (99.7)	
Yes	93 (97.9)	213745 (99.6)		2 (2.2)	666 (0.3)	
Carbonate beverages			0.683			0.690
Never	58 (61.1)	129665 (60.4)		57 (63.3)	124338 (57.9)	
Always	0	2578 (1.2)		1 (1.1)	2188 (1.0)	
Often	15(15.8)	28821 (13.4)		11 (12.2)	29660 (13.8)	
Occasionally	13 (13.7)	26871 (12.5)		13 (14.4)	29093 (13.5)	
Seldom	9(9.5)	26566 (12.4)		8 (8.9)	29595 (13.8)	
Coffee			0.626			0.661
No	88 (92.6)	200608 (93.5)		85 (94.4)	200447 (93.3)	
Yes	7 (7.4)	13893 (6.5)		5 (5.6)	14427 (6.7)	
Tea			0.444			0.736
Never	51 (53.7)	98910 (46.1)		56 (62.2)	137557 (64.0)	
Always	26 (27.4)	68840 (32.1)		15 (16.7)	34585 (16.1)	
Often	6 (6.3)	22790 (10.6)		7 (7.8)	18266 (8.5	
Occasionally	7 (7.4)	14672 (6.8)		5 (5.6)	14242 (6.6)	
Seldom	5 (5.3)	9289 (4.3)		7 (7.8)	10224 (4.8)	
House facing the sun			0.05			0.984
No	3 (3.2)	2314 (1.1)		1 (1.1)	2335 (1.1)	
Yes	92 (96.8)	212187 (98.9)		89 (98.9)	212439 (98.9)	
Living circumstance			0.800			0.832
Single-storey house	41 (43.2)	85619 (39.9)		36 (40.0)	84696 (39.4)	
House <=7 storey	47 (49.5)	113177 (52.8)		46 (51.1)	114358 (53.2)	
House >7 storey	7 (7.4)	15705 (7.3)		8 (8.9)	15820 (7.4)	
Calcium or Vitamin D supplement			0.700			0.976
No	91 (95.8)	203608 (94.9)		84 (93.3)	200715 (93.4)	
Yes	4 (4.2)	10893 (5.1)		6 (6.7)	14159 (6.6)	
Average sleep time (hours) per day			0.003			0.001
≥7	49 (51.6)	141352 (65.9)		47 (52.2)	76014 (35.4)	
<7	46 (48.4)	73149 (34.1)		43 (47.8)	138860 (64.6)	
Previous history of fracture			0.034			<0.001
No	89 (93.7)	208585 (97.2)		83 (92.2)	211081 (98.2)	
Yes	6 (6.3)	5916 (2.8)		7 (7.8)	3793 (1.8)	
Menopause (age, year)						<0.001
<46				1 (1.1)	5366 (2.5)	
46–50				41 (45.6)	57310 (26.7)	
>50				12 (13.3)	19301 (9.0)	
Pre-menopausal				36 (40.0)	132897 (61.8)	
Children to give birth						<0.001
No				9 (10.0)	33559 (15.6)	
1				18 (20.0)	82164 (38.2)	
2				47 (52.2)	68588 (31.9)	
3				13 (14.4)	23874 (11.1)	
≥4				3 (3.3)	6689 (3.1)	

Table 5 summarized independent risk factors for traumatic ankle fractures in adults by gender. For males, alcohol consumption and living alone increased the risk of ankle fracture by 1.86 times (95% CI, 1.21–2.88) and 5.05 times (95% CI, 1.23–20.76), respectively. And compared to those having enough sleep time (>=7 h/d), average sleep time <7 h/d increased the risk of ankle fracture by 1.72 times (95% CI, 1.15–2.57). Housing facing the sun seemed to have protective effect on ankle fracture, but the significance did not approach to statistical level (P = 0.074). For females, alcohol consumption, living alone and average sleep time <7 h/d were identified as independent risk factors for ankle fracture and the corresponding ORs were 3.00 (95% CI, 1.87–4.79), 5.93 (1.45–24.30) and 1.63 (95% CI, 1.06–2.50). Previous history of fracture was identified as a significant risk factor in females (OR, 3.68; 95% CI, 1.69–8.03) but not in males (OR, 2.13; 95% CI, 0.93–4.88).

**Table 5:** Results of multivariate logistic regression analysis of risk factors for ankle fractures.

Variables	Exp (B)	95% CI		*P*
		Lower limit	Upper limit	
Males				
Alcohol consumption	1.863	1.207	2.875	0.005
Living alone	5.052	1.229	20.762	0.025
House facing the sun	0.336	0.106	1.071	0.065
Sleep time <7 h/d	1.718	1.148	2.571	0.009
History of previous fracture	2.132	0.930	4.883	0.074
Females				
Alcohol consumption	2.995	1.871	4.794	<0.001
Living alone	5.927	1.446	24.303	0.013
Sleep time <7 h/d	1.629	1.060	2.500	0.026
History of previous fracture	3.682	1.688	8.029	0.001

In the final multivariate logistic regression model, the Hosmer–Lemeshow test demonstrated the adequate fitness either for males (X^2^ = 1.510, P = 0.680) or females (X^2^ = 8.006, P = 0.433).

## Discussion

In the present study, data from CNFS database of traumatic fractures in China showed incidence rate of ankle fracture was 37.1/100,000 person-year in 2014. Results also showed 76.8% of ankle fractures were caused by slip, trip or fall, and 72.1% of all ankle fractures occurred at home and on the road around. In adults, alcohol consumption, living alone and average sleep time <7 h/d significantly increased the risk of ankle fractures, either in males or females. Females with previous history of fracture of any site had the 3.68-time increased risk of ankle fractures (P < 0.001), but for males the significance did not approach to statistical level (P = 0.074).

So far as known, this is currently the most comprehensive and detailed epidemiologic survey based on questionnaires, for investigation of the population-based incidence of ankle fractures and associated risk factors. In 2017, data on traumatic fractures of the trunk, arms, or legs (not including the skull, sternum, and ribs) that had occurred in 2014 were published^8^. In that study^8^, we reported the national population-based incidence rates of fractures, based on sites, age, sex and others (ethnic origin, occupation, geographical region, and residency category) and identified some risk factors for the overall fractures based on age groups (<15, 15–64, and ≥65 years). In the current study, we only focused on ankle fractures and reported their population-based incidence, injury mechanism, place of fracture occurrence and identified some associated risk factors, which was of more pertinence in knowledge and prevention of these injuries.

Compared to the previous reports, the incidence rate of ankle fractures was considerably lower in this study. The highest incidence rate was reported by Daly *et al*.^4^, who used an estimated population size and reported the incidence rate of ankle fractures was 187/100,000/persen-year during 1979–1981 in Rochester, Minnesota. Elose *et al*.^2^ reported the incidence of 168.7/100000/person-year, with data from 9767 ankle fracture patients of all age in a Denmark university hospital. Court-Brown *et al*.^10^ reported an overall incidence of 100.8/100000/year in 2000 and 13.7/100000/year in 2014 In Edinburgh, wherein all patients were above 15 years. Thur *et al*.^11^ reported the incidence of 71/100000/year in a population-based study of 91,410 Swedish inpatients during 1987–2004 years, with outpatients and patients <15 years excluded. Jensen *et al*.^6^ conducted a prospective population-based study of 212 cases of ankle fractures in a population about 200,000 in Aalborg, Denmark, and reported the overall incidence of 107 per 100,000 person-years. However, in their study, all the ankle fracture patients were admitted in the emergency department^6^. The great variation in incidence rate of ankle fracture reported in literature may be due to the differences in geographic locations or lifestyles differences, time periods, population sizes and the exclusion of certain patients. In contrast, the present study was conducted with a more accurate population size and all fracture cases were initially confirmed by patients’ self-reports and further confirmed by medical data. Therefore, it should make more sense on estimation of overall ankle fractures in China.

It is accepted that the most common mechanism causing ankle fractures was low-energy trauma^2,10–12^. In this study, we found the similar result that over three quarters of ankle fractures were caused by slip, trip or fall from standing height. Elsoe *et al*.^2^ suggested sports (22%) and Thur^11^ *et al*. suggested fall from height (10%) was the second most common injury mechanism leading to ankle fractures, which was different from our result (traffic injuries, 11.6%). Primarily, the difference was due to the different life and recreation styles among different regions and countries. In addition, we found 72.1% of all ankle fractures occurred at home and on the road around. Therefore, primary prevention including home prevention remains the major task for reduction of ankle fractures, especially for elderly individuals.

In the present study, alcohol consumption, living alone and sleeping time less than 7 h per day was identified as independent risk factor for ankle fractures for adults, either for males or females. Alcohol consumption as a recognized risk factor for traumatic fracture had been identified in the literature^12,13^. It was reported that consuming more than 8 units of alcohol for men or more than 6 units for women in the past week significantly increased the risk of fractures in individuals aged 55 years and older^12,13^. And the underlying mechanism might be metabolic effects and alcohol-related falls^13^. Stone *et al*.^14^ reported that women who slept for 5 h or less or 5–7 h had the higher risk of frequent falls, compared to those with adequate sleep (7–8 h/d). And Holmberg *et al*.^15^ got the similar finding in males that sleep disturbances contributed to the increased risk in most fractures of bone sites, including ankle fracture. Lazkani and his colleagues^16^ identified living alone as an independent risk factor for recurrent falls in elderly individuals, and this finding was also observed in other studies^17–19^. In addition, living alone had been identified to be associated with depressive symptoms^20^, self-neglect^21^, and physical inactivity^22^, all of which could exert negative effects on the ankle fractures. Therefore, education on healthy lifestyles should be advocated and for individuals living alone, and anti-fall measures should be taken especially at their home and if necessary, mental consolation could be implemented.

In the present study, previous history of fracture was identified as an independent risk factor for females and increased 3.68-time risk of ankle fracture, and marginally significant for males (P = 0.074). Similarly as ours, Holmberg and colleagues^15^ reported that previous low-energy fractures strongly increased the risk of subsequent fracture in middle-aged women, but not in men. However, another study by Klotzbuecher *et al*.^23^ showed that elderly patients either males or females with prior fracture had increased risk of subsequent fracture. Gunnes *et al*.^24^ conducted a questionnaire study of 29,802 postmenopausal women and found that patients with prior fractures had the increased risk of ankle fractures by 1.6-time. Therefore, we suggested the history of previous could have negative effects on both males and females; and compared to males, females were more likely to be influenced at some certain fractures, such as ankle fracture. It was notable that, this conclusion was drawn in the context of limited cases of ankle fractures and should be treated cautiously, and required further studies to confirm.

Although being the largest questionnaire survey currently, this study had some potential limitations. Firstly, the retrospective nature of this study had its intrinsic weakness in accuracy of data collection. Secondly, the results of patients’ self-report on fracture occurrence and individual life styles might be affected more or less. For example, the specificity of detection fracture cases using patients’ self-report and further confirmation by medical data could be up to 100%, but the sensitivity might be not so high, because in some patients they choose to evade injuries for individual reason. Thirdly, there might be some selection bias, as we could not capture data about traumatic ankle fracture or concurrent visceral injury in which the individual had died. Therefore overall, the incidence rate of traumatic ankle fracture was underestimated.

In summary, the current study provided detailed information about the national population-based incidence, characteristics and related risk factors of ankle fractures, which should be of great importance in national healthcare planning and individual health consultation. Specific public health policies focusing on decreasing alcohol consumption and encouraging individuals to obtain sufficient sleep should be implemented. Individuals living alone should focus on more on healthcare, active exercises and maintaining good mental status, especially in those with history of previous fracture.
